# Mature Luffa Leaves (*Luffa cylindrica* L.) as a Tool for Gene Expression Analysis by Agroinfiltration

**DOI:** 10.3389/fpls.2017.00228

**Published:** 2017-02-21

**Authors:** Kamila Błażejewska, Małgorzata Kapusta, Elżbieta Zielińska, Zbigniew Tukaj, Izabela A. Chincinska

**Affiliations:** ^1^Department of Plant Physiology and Biotechnology, Faculty of Biology, University of GdańskGdańsk, Poland; ^2^Department of Plant Cytology and Embryology, Faculty of Biology, University of GdańskGdańsk, Poland

**Keywords:** cucurbits, agroinfiltration, luffa, phloem exudate, plant overexpression system, foliar application of tracers

## Abstract

We exploited the potential of cucurbits for ectopic gene expression. Agroinfiltration is a simple and commonly used method to obtain transient expression of foreign genes in plants. In contrast to *in vitro* transformation techniques, agroinfiltration can be used for genetic modification of mature plant tissues. Although the cucurbits are commonly used as model plants for molecular biology and biotechnology studies, to date there are no literature sources on the possibility of transient gene expression in mature cucurbit tissues. Our research has shown that mature leaves of *Luffa cylindrica* L. (luffa), in contrast to other cucurbit species, can be successfully transiently transformed with *Agrobacterium tumefaciens.* We efficiently transformed luffa leaves with a reporter gene encoding β-glucuronidase (GUS). The GUS activity in transiently transformed leaf tissues was detected within 24 h after the infiltration with bacteria. Additionally, we have shown that the activity of a transiently expressed the GUS gene can be monitored directly in the EDTA-exudates collected from the cut petioles of the agroinfiltrated leaves. The results suggest that luffa leaves can be useful as a plant expression system for studies of physiological and biochemical processes in cucurbits.

## Introduction

The *Cucurbitaceae* species (cucurbits) are a significant source of food and substances of medical importance (Manamohan et al., 2011). Several species of *Cucurbitaceae*, for example *Cucurbita maxima, Cucurbita pepo, Cucurbita ficifolia* or *Cucumis sativus*, are widely used as model plants for research, especially in studies of phloem functions and biochemistry (Golecki et al., 1999; Zhang et al., 2010, 2012).

The use of cucurbits in phloem research was primarily associated with the ease of exudate sampling from severed stems and petioles (Richardson et al., 1982; Lin et al., 2009). The fluid exuding from the cucurbit vascular tissue contains phloem sap derived from the fascicular phloem as well as from the extrafascicular phloem (Zhang et al., 2010, 2012; Zimmermann et al., 2013). This is a spatially distinct system of sieve elements occurring specifically in *Cucurbitaceae* species (Turgeon and Oparka, 2010). Cucurbit exudates are rich in proteins (their concentration can reach up to 60–100 mg/mL) (Richardson et al., 1982; Walz et al., 2004). It was shown, that the exudate proteins applied to *C. maxima* cotyledons trafficked symplastically through mesophyll plasmodesmata and translocate over long distance in the phloem (Balachandran et al., 1997). Various techniques, like the intergeneric grafting experiments or the studies on systemic movement of plant virus particles and phloem tracers, e.g., 5(6)-carboxyfluorescein or fluorescein diacetate, were used to investigate the phloem loading mechanisms and the long-distance trafficking of molecules in cucurbits (Grignon et al., 1989; Golecki et al., 1999; Pradel et al., 1999; Zhang et al., 2010). To date, there are only a few reports in which transgenic cucurbit species were used for investigation of phloem function. In contrast, the techniques enabling the induction of heterological expression in plant tissues are widely used as research tools for phloem studies of many non-cucurbit species, especially the plants defined as apoplastic loaders (Chincinska et al., 2008; Krügel et al., 2012).

Agroinfiltration belongs to the most popular plant transformation techniques. In this method, the suspension of *Agrobacterium*, carrying a binary vector with a targeted transgene, is pressed into the intercellular spaces of a selected plant tissue, usually a leaf. Thus, the successful introduction of bacterial suspension is one of the most important requirements affecting the efficiency of transient transformation using agroinfiltration (Bhaskar et al., 2009; Leuzinger et al., 2013). The agroinfiltration technique was well-developed for model plants such as *Nicotiana benthamiana* (Wydro et al., 2006; Goodin et al., 2008), *Nicotiana tabacum* or *Arabidopsis thaliana* (Cazzonelli and Velten, 2006; Kim et al., 2009). This method was also applied in several crops, e.g., tomato (*Lycopersicon esculentum* Mill.), lettuce (*Lactuca* L.) (Joh et al., 2005), potato (*Solanum tuberosum* L.) (Bhaskar et al., 2009), and grape (*Vitis* L.) (Santos-Rosa et al., 2008). Nevertheless, to date, there is no agroinfiltration protocol for generation of plant expression systems based on mature cucurbit tissues. Therefore, the potential application of various cucurbit species for a gene functions assay using agroinfiltration was tested in this study. We showed that mature leaves of *Luffa cylindrica* L., in contrast to other cucurbits tested, can be efficiently agroinfiltrated. We detected the expression of a gene encoding GUS driven by the CaMV 35S promoter in luffa leaves a few hours after agroinfiltration. Moreover, the GUS activity was also detected in the EDTA-exudates collected from the cut petioles of the transiently transformed luffa leaves.

## Materials and Methods

### Plant Material

The *Cucurbitaceae* species were cultivated from seeds (Legutko Breeding and Seed Company, Ltd) and grown in a greenhouse in 30 cm diameter pots. All plants were grown at 270 μmol photons/m^2^/s with a light/dark cycle of 16 h/8 h at 25/16°C and 40–50% relative humidity.

### Microscopy

Parts of leaf blades of examined cucurbit species were fixed in 8% formaldehyde and 0.25% glutaraldehyde in piperazine buffer at 4°C overnight then embedded in Steedman’s Wax and sectioned at 10 μm (Krawczyk et al., 2016). The chromatin of the nuclei was visualized with 1 μg/mL Propidium Iodide and cell walls were stained with 0.1% Calcofluor White M2R. Specimens were closed in PBS buffer and viewed in epifluorescence with Leica DM6000 B supported by LAS AF software. Images of leaf blades are maximum projections of taken Z-stacks, deconvolved using five iterations of a 3D Non-blind algorithm (AutoQuant^TM^) to maximize spatial resolution.

### Agroinfiltration Procedures

For agroinfiltration analysis in luffa plants we used *Agrobacterium tumefaciens* strain LBA 4404 transformed with a commercially available vector pRI 201-AN-GUS (Takara, Clontech Laboratories, Inc.) containing β-glucuronidase gene (*uidA*) sequence under the control of the CaMV 35S promoter. The untransformed *A. tumefaciens* strain LBA 4404 was used as a negative control. The *Agrobacterium* pre-culture conditions and the preparation of the bacteria suspension were prepared according to the procedure described previously and optimized for agroinfiltration of *N. benthamina* (Wydro et al., 2006). Mature leaves were harvested from the luffa plants growing in the greenhouse. The leaves were cut at the petiole bases, then transported to the laboratory and stored in a closed container. High humidity in the containers was held by placing wet paper towels at the bottom. Immediately before the infiltration the leaf was weighed, then placed adaxial side down on a layer of soft paper towels designed to protect the leaf from mechanical damage during the syringe infiltration. Subsequent fragments of the leaf were infiltrated using a 1 mL syringe. The leaf saturated with the bacterial suspension was gently dried with a paper towel and weighed. Immediately after infiltration the leaves were placed in plant propagators (the dimensions: 58 cm × 40 cm × 22 cm, Supplementary Figure S1). To maintain the optimal physiological condition of the leaves as well as to stabilize their turgor, propagator bottoms were coated with a 1 cm thick layer of water and covered with clear plastic covers. The propagators with leaves were then placed under the LED lamps.

### Phloem Exudation

The petioles of the leaves previously infiltrated with the bacteria as well as the control leaves were submersed under 2.5 mM EDTA solution (to prevent the contact with atmospheric oxygen) and recut by 2–3 mm. The leaves were immediately transferred to dark plastic tubes containing 5 mL of 2.5 mM EDTA solution to facilitate exudation. To protect against evaporation of EDTA solution the tubes were carefully sealed with parafilm.

### Protein Analysis

Leaves without petioles were ground in 30 mL of protein extraction buffer [50 mM NaHPO4, pH 7.0, 10 mM β-mercaptoethanol, 10 mM EDTA, 0.1% (w/v) sodium lauryl sarcosine, and 0.1% (w/v) Triton X-100]. The leaf extracts and the EDTA-exudates were used for the total soluble protein (TSP) content measurement using the Bradford (1976) method and for GUS activity assays.

### Electrophoresis and Western Blotting

SDS-PAGE was performed according to Brunelle and Green (2014), with the modification that acrylamide/bisacrylamide solution mix was in ratio (29:1) in both stacking and resolving gels. Exudate samples were separated in a 12% polyacrylamide gel and stained with Coomassie brilliant blue. Western blotting was performed as described previously (Brunelle and Green, 2014). The anti-β-glucuronidase (C-Terminal) primary antibody (Sigma–Aldrich) and the goat anti-IgG rabbit coupled with HRP (horseradish peroxidase) secondary antibody were used for GUS detection. A recombinant GUS protein from *Escherichia coli* (Sigma–Aldrich) was used as a reference.

### GUS Activity Analysis

The histochemical GUS staining and fluorogenic GUS assays were performed as described by Jefferson et al. (1987), using the 5-bromo-4-chloro-3-indolyl glucuronide (X-gluc) and 4-methylumbelliferyl-β-D-glucuronide hydrate (4-MUG) (Bio Basic, Inc.) as substrates, respectively. Because the fluorescence intensity of 4-methylumbelliferone (4-MU) is influenced by the presence of plant extracts, the 4-MU concentration curves (4-MU standard curves) for the GUS activity assays were generated separately in the exudates and in the leaf extracts using the leaf extracts and exudates, respectively, for the standard curve preparation. Spectrophotometric and fluorescence measurements were done using Varioscan Flash Multimode Reader (Thermo Scientific).

## Results

### Leaves of Different Cucurbit Species Show Different Infiltration Susceptibility

We selected 11 different cucurbit species (**Table 1**) to test a susceptibility of their leaves to infiltration. The mature leaves (source leaves from the middle part of the stem) were collected from 2-month old plants and then infiltrated with 1 mL pure infiltration medium to test their absorption properties. We anticipated that the difference in the leaf weight before and after infiltration would correspond to the volume of resuspension medium absorbed by the leaf tissue. The infiltration area, visible after the injection as a darker stain on the leaf surface, was also evaluated (**Table 1**). The results showed that *L. cylindrica* leaves could absorb significantly more fluid than the other cucurbits. A single application of the medium into the luffa leaf covered an average leaf area of 8.22 ± 1.23 cm^2^ (± SD; *n* = 8) and was significantly higher in comparison to all other species. Because the fluid introduced into the luffa leaves was easily absorbed and quickly diffused in the adjacent tissue around the injection site, it became possible after a few injection repetitions to bring the leaves to the complete saturation with the fluid.

**Table 1:** Comparison of cucurbit leaves absorption capacity.

	Difference in the leaf weight before and after infiltration				Infiltration surface area					
Cucurbit varieties	Mean [g]	*SD*	Homogenous groups		Mean [cm^2^]	*SD*	Homogenous groups			
			a	b			a	b	c	d
*Cucurbita maxima* Duch. cv. Bambino	0.097	0.070	^∗^		0.47	0.12	^∗^			
*Cucurbita pepo* L. cv. Makaronowa Warszawska	0.138	0.115	^∗^		0.48	0.13	^∗^			
*Cucurbita maxima* Duch. cv. Melonowa Żółta	0.096	0.025	^∗^		0.64	0.96	^∗^			
*Lagenaria sinceraria* Standl. cv. Kobra	0.139	0.040	^∗^		0.88	0.18	^∗^			
*Cucumis metuliferus* Mey.	0.060	0.052	^∗^		1.00	1.05	^∗^			
*Lagenaria sinceraria* Standl. cv. Birdhouse	0.179	0.122	^∗^		1.17	2.81	^∗^			
*Cucurbita moschata* Duch.	0.149	0.091	^∗^		1.36	0.44	^∗^	^∗^		
*Cucurbita maxima* Duch. cv. Atlantic Giant	0.168	0.138	^∗^		1.52	0.57	^∗^	^∗^		
*Lagenaria sinceraria* Standl. cv. Marenka	0.218	0.099	^∗^	^∗^	3.24	0.49		^∗^	^∗^	
*Lagenaria sinceraria* Standl. cv. Snake	0.113	0.039	^∗^		4.24	2.23			^∗^	
*Luffa cylindrica* L.	0.354	0.221		^∗^	8.22	1.23				^∗^

*We measured the amount of the medium absorbed by the leaf and the infiltration surface area. The leaves were infiltrated with 1 mL pure infiltration medium. One-way ANOVA and the Scheffé’s *post hoc* method was used for statistical analysis. Homogeneous groups are marked with asterisks under the same letter. Total *n* = 6 leaves from at least two individual plants of each species were used. The experiment was repeated two times independently.*

A relatively high leaf absorption capacity was also shown in the case of the *Lagenaria* species. However, the infiltration of *Lagenaria* leaves proved to be very time consuming due to the delicate structure of their leaves. The infiltration of *Lagenaria* leaves required very careful handling to prevent mechanical damage of the tissues while the luffa leaves showed relative high resistance to damage. In contrast, the infiltration of the mature leaves of *Cucurbita* and *Cucumis* species appeared to be extremely difficult.

### Luffa Leaves Anatomy Can Explain their Excellent Absorptivity

**FIGURE 1:**
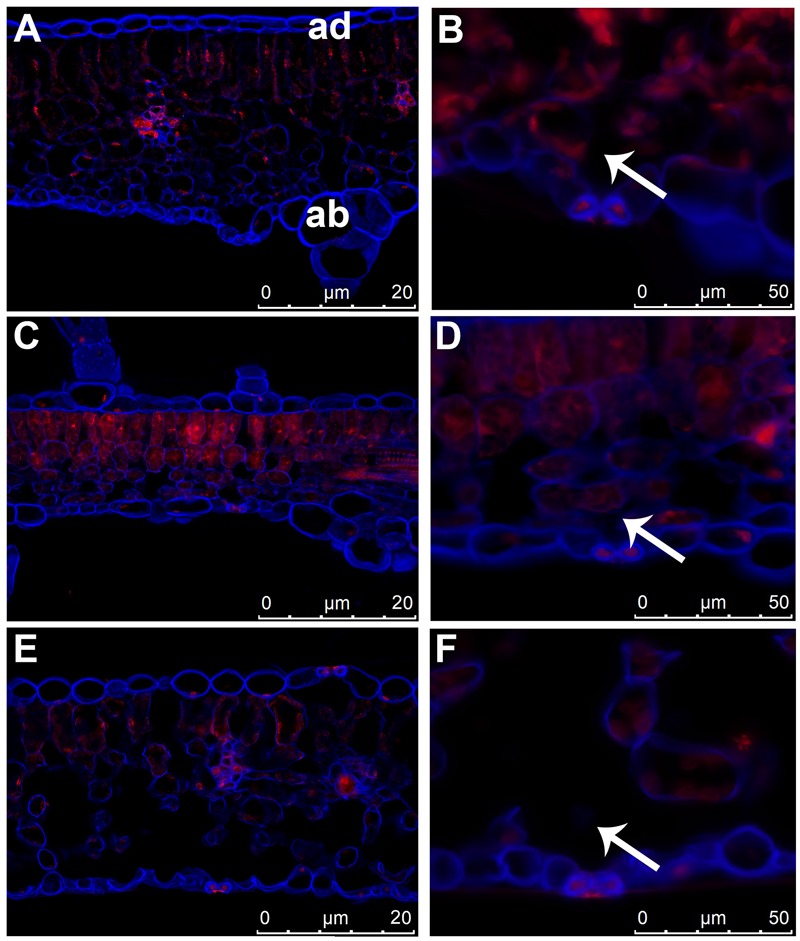
**Differences in the leaf structure of cucurbit species.** Leaf cross sections of three cucurbit species stained with Calcofluor White and propidium iodide. **(A,B)** *Cucurbita pepo* L. cv. Makaronowa Warszawska. **(C,D)** *Cucurbita moschata* Duch. **(E,F)** *Luffa cylindrica* L. **(A,C,E)** anatomical comparison of cucurbit leaves showed large air spaces in luffa leaf mesophyll **(E)**, while the mesophyll cells in leaves of both *Cucurbita* species were closely packed **(A,C)**. The 3D projections were acquired from z-stacks of cross sectioned leaves. ad = adaxial, ab = abaxial part of leaf in **(A)** corresponds to all photos. **(B,D,F)** stomata structure in cucurbit leaves is similar in all species analyzed, but the sub-stomatal air spaces (marked with an arrow) is significantly greater in luffa leaf **(F)** than in the both cucurbit **(B,D).**

Structure of leaf tissues, especially cuticle thickness, stomata anatomy and density, significantly affect the susceptibility of various plant species to foliar infections by pathogenic bacteria (Melotto et al., 2008). Since the agroinfiltration procedure involves injection of bacteria suspension into the air spaces of leaf mesophyll, it can be assumed that these leaf structures have a significant impact on the introduction of bacteria by agroinfiltration. We used epifluorescence microscopy to compare the anatomical structure of luffa leaves (**Figure 1E**) with the leaves of two selected *Cucurbita* species (*C. pepo* L. cv. Makaronowa Warszawska and *C. moschata* Duch), recognized previously as difficult to infiltrate (**Table 1**; **Figures 1A,C**). We observed large air spaces in the luffa leaf mesophyll, while the mesophyll cells in leaves of both *Cucurbita* species were closely packed. The analysis revealed no differences between the structure of the cuticle and the guard cells in the analyzed leaves, but the air spaces located directly behind the stomata in luffa leaves (**Figure 1F**) were significantly larger than in the *Cucurbita* leaves (**Figures 1B,D**). This looser structure, in particular the extensive stomata air spaces, certainly affects the susceptibility of luffa leaves to the bacteria injection.

### Agroinfiltration of Luffa Leaves

Because luffa stems achieve a length of up to 10 m under greenhouse conditions and produce a large number of leaves, it was not possible to agroinfiltrate the plants completely which is a common practice in the case of *N. benthamiana* (Leuzinger et al., 2013). Therefore, we decided to infiltrate the individual leaves cut from the donor plants. The average area of the mature leaves (harvested from the middle part of the stems) was 203.5 ± 84.3 cm^2^ (± SD; *n* = 116). The leaves were treated with *A. tumefaciens* strain LBA 4404 transformed with the binary vector pRI 201-AN-GUS carrying *E. coli uidA* gene encoding GUS. As a control, we used luffa wild type (wt) leaves, untreated with the bacteria or the leaves infiltrated with *A. tumefaciens* LBA 4404 without the binary vector. The bacteria suspension was prepared according to the standard protocol widely used for *N. benthamiana* agroinfiltration (Wydro et al., 2006). To prevent the loss of turgor, the leaves immediately before and after agroinfiltration were kept under high humidity conditions (Supplementary Figure S1).

We observed that the leaves incubated in high humidity conditions showed excellent susceptibility to the infiltration for many hours after harvesting. Moreover, the introduction of bacteria (OD_600_ = 1.0) into the leaves stored in high humidity was much easier than in the case of leaves that had recently been cut from the host plants. The increased agroinfiltration efficiency at high humidity growth conditions was reported previously in *Arabidopsis* (Kim et al., 2009). High humidity promotes the opening of the stomata, which in consequence, increases the access of the externally applied fluid to the sub-stomatal air spaces, as well as to the air spaces located in the deeper parts of the spongy and palisade mesophyll. The increased humidity conditions did not significantly affect the agroinfiltration susceptibility of the other cucurbit species we tested. We suppose, that the compact structure (**Figure 1**) of the leaf tissues in many cucurbit species efficiently prevents the penetration of the externally applied liquids into the deeper tissues despite the stomata opening.

**FIGURE 2:**
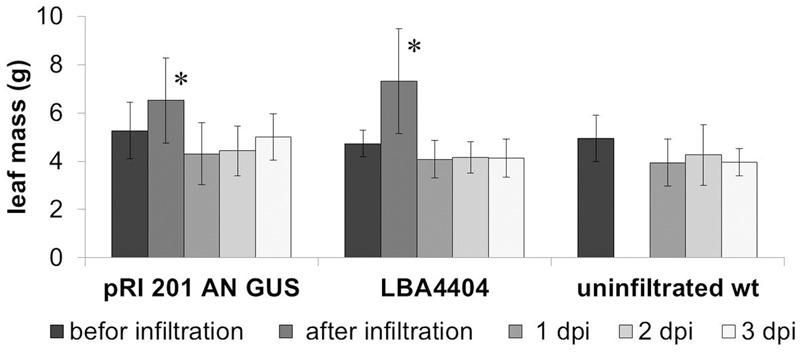
**Changes in the weight of agroinfiltrated leaves.** The average weight of the leaves infiltrated with *Agrobacterium tumefaciens* carrying pRI 201-AN-GUS (*n* > 6) as well as the leaves infiltrated with *A. tumefaciens* (*n* = 6) without binary vector increased significantly directly after introduction of bacteria suspension (marked with an asterisks; *P* < 0.01) but at 1 dpi decreased the average weight significantly and then remained stable in the consecutive days after infiltration. The analysis was repeated independently three times.

The leaves were weighed before and immediately after the infiltration to estimate the amount of the bacterial suspension absorbed. The biomass of leaves was also measured up to three post-infiltration days to monitor a turgor loss in the leaves. Directly after saturation of the leaf with the bacterial suspension, the average mass of luffa leaves increased by 22.7 ± 12.4% (± SD; *n* = 14), relative to the leaf biomass before the agroinfiltration (**Figure 2**). However, 1 day post-infiltration (1 dpi) the mass of the infiltrated leaves decreased significantly, returning to the values similar to those measured immediately before the infiltration. The leaf mass then remained stable for at least 3 days (2 and 3 dpi) (**Figure 2**). The comparison of the biomass changes in the wt leaves with the leaves infiltrated with the bacteria suspension (*A. tumefaciens* with or without the binary vector) in the days following their harvesting from the donor plants, demonstrates that the infiltration procedures we used are insignificant for the transpiration intensity in luffa leaves (**Figure 2**).

### GUS Activity in Leaves and Exudates

**FIGURE 3:**
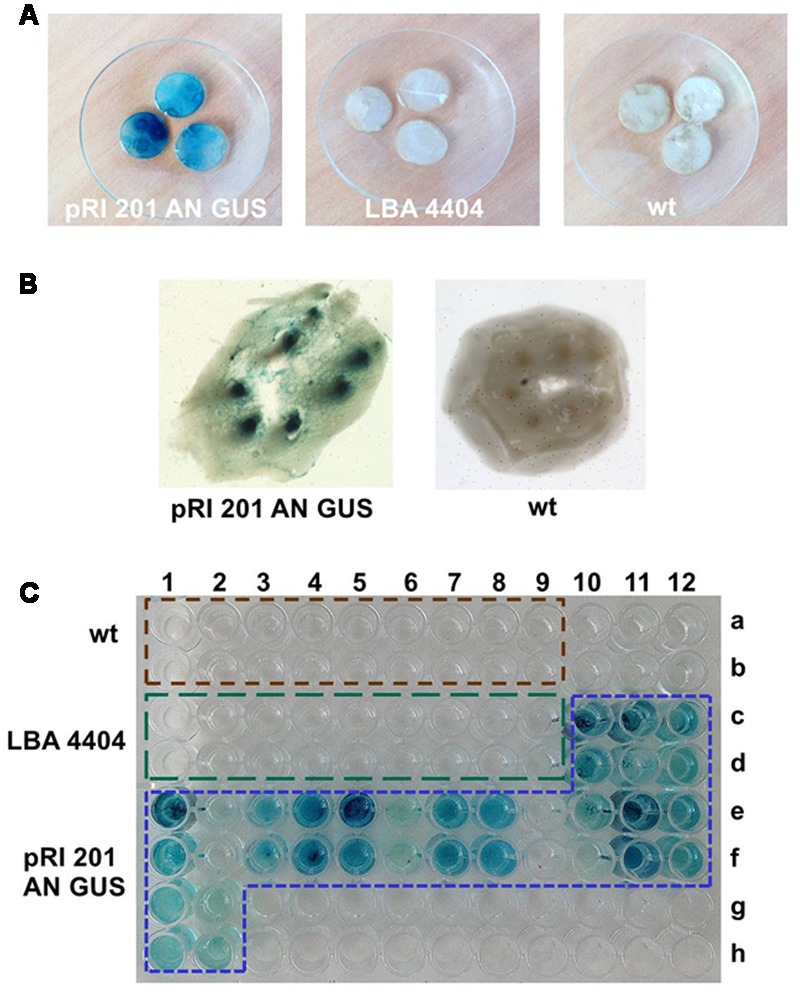
**The histochemical X-gluc staining of the GUS activity in luffa leaves and EDTA-exudates. (A)** Leaf disks (10 mm in diameter) randomly collected from: the leaves infiltrated with *A. tumefaciens* carrying pRI 201-AN-GUS, the leaves infiltrated with *A. tumefaciens* LBA 4404 without the binary vector, wt leaves. **(B)** Petiole cross section of the leaf infiltrated with *A. tumefaciens* carrying pRI 201-AN-GUS and from luffa wt leaf. The blue stained vascular bundles are shown. **(C)** GUS activity in EDTA-exudate samples detected with X-gluc on microtiter plate. The 100 μL samples were collected at 1 dpi from wt leaves (wells A1–A9 and B1–B9), from the leaves infiltrated with untransformed *A. tumefaciens* LBA 4404 (wells C1–C9 and D1–D9) and from the leaves infiltrated with *A. tumefaciens* carrying pRI 201-AN-GUS (wells C10–C12, D10–D12, E1–E12, F1–F12, G1–G2, H1–H2). The histochemical analysis of leaf tissues and exudates were repeated independently at least three times.

The histochemical detection revealed the high GUS activity in all random samples collected from the leaves agroinfiltrated with the vector pRI 201-AN-GUS, whereas the wt leaves and the leaves infiltrated with *Agrobacterium* without the binary vector did not show the GUS activity (**Figure 3A**, Supplementary Figure S2). The staining pattern in the leaf fragments was non-uniform indicating the heterogeneous GUS activity in the agroinfiltrated tissues. Therefore, we decided to sample the whole leaf blades for further GUS analysis. Fluorometric assays estimated the GUS activity in the extracts from the whole pRI 201-AN-GUS transformed leaf blades as 31.7 ± 13.2 (± SD; *n* = 6); 34.2 ± 16.9 (± SD; *n* = 6) and 50.4 ± 27.4 (± SD; *n* = 5) [μM 4-MU/mg total soluble proteins/min], at 1, 2, and 3 dpi, respectively. When the GUS activity in leaves extracts was normalized to the total surface area of the infiltrated leaves the following values were received: 720.9 ± 258.6 (± SD; *n* = 6); 823.0 ± 325.7 (± SD; *n* = 6) and 874.6 ± 557.4 (± SD; *n* = 5) [μM 4-MU/min in 100 cm^2^ of the leaf surface], at the 1, 2, 3 dpi respectively. The statistical analysis showed no significant differences between the GUS activity in the leaves measured at 1, 2, and 3 dpi. However, the fluorescence measured in the presence of MUG-substrate in the transiently transformed tissues was significantly higher in comparison to the fluorescence of the controls (wt and the leaves infiltrated with *Agrobacterium* without the binary vector).

**FIGURE 4:**
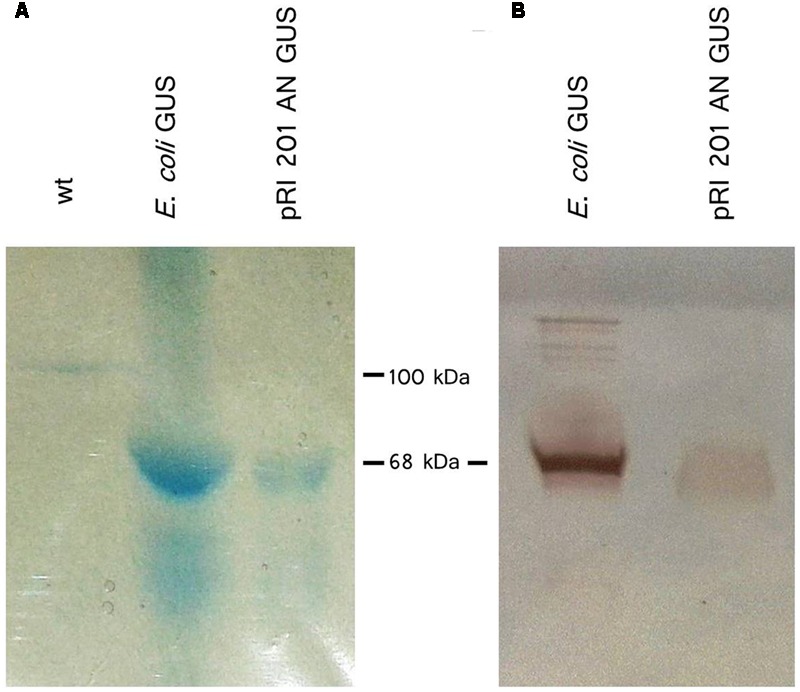
**β-Glucuronidase (GUS) protein detection in EDTA-exudates. (A)** Exudates from petioles of wt luffa leaf (lane 1; 41 μg total protein) and from the leaf infiltrated with *A. tumefaciens* carrying the pRI 201-AN-GUS (lane 2; 51 μg total protein) were separated by SDS-PAGE and stained with Coomassie Blue. **(B)** Western blot analysis of exudate from pRI 201-AN-GUS agroinfiltrated leaf (lane 1; 30 μg total protein). 1 μg of GUS from *E. coli* was used as a reference.

The GUS activity was detected not only in agroinfiltrated leaf blades but also in vascular bundles in the petioles (**Figure 3B**) indicating the presence of active GUS (tetrameric form) in the tissues distant from the *Agrobacterium* introduction. Also, exudates sampled directly to 2.5 mM EDTA-solution (King and Zeevaart, 1974) from cut petioles of agroinfiltrated leaves were stained blue in the presence of X-gluc (**Figure 3C**). We analyzed exudate samples by SDS-PAGE and Western blot (**Figure 4**). The band with expected protein mass of 68 kDa corresponding to the *E. coli* GUS protein standard (monomeric form) was detected only in the exudate sampled from transiently transformed leaves.

In order to evaluate the differences between the GUS activity in the transformed leaf tissues and the GUS activity in the EDTA-exudates collected from these leaves, we performed the fluorescence assays in four independent experiments (Experiments I–IV, **Table 2**). The GUS activity was generally normalized to the TSP concentration. However, due to the large differences in the TSP content in leaf extracts and in exudates, the method was inappropriate for the comparative analysis of the GUS activity. Therefore, the GUS activity values were also normalized to the total surface area for the comparison analysis (**Table 2**; Supplementary Table S1). Despite the dispersion of the obtained results, the statistical analysis showed clearly that the EDTA-exudates as well as the leaves extracts collected from the transiently transformed tissues, exhibited significantly higher fluorescence in the presence of MUG, than the samples collected from the control leaves.

**Table 2:** The GUS activity in the agroinfiltrated luffa leaves and exudates.

GUS activity [μM 4-MU/min/100 cm^2^ leaf]												
				**EDTA-exudates**								
**No. experiment**	**Leaf extracts**			**1 dpi**			**2 dpi**			**3 dpi**		
	**Mean**	***SD***	***n***	**Mean**	***SD***	***n***	**Mean**	***SD***	***n***	**Mean**	***SD***	***n***

Experiment I	79.8	58.5	6	60.4	36.5	6	63.5	38.4	6	7.7	2.3	6
Experiment II	244.2	146.3	10	87.2	61.8	4	14.9	8.5	4	10.3	14.6	4
Experiment III	*nc*	*nc*	*nc*	164.5	64.7	4	24.8	11.9	4	27.0	17.3	4
Experiment IV	710.4	369.3	14	50.3	54.5	14	*nc*	*nc*	*nc*	*nc*	*nc*	*nc*

*The GUS activity were measured fluorescently in leaves extracts and EDTA-exudates and then normalized to the 100 cm^*2*^ leaf surface area. In the Experiments I–II the leaves extracts were collected at 3 dpi and in the Experiment IV at 1 dpi. In the Experiments I–III the exudates were collected at 1–3 dpi and in the Experiment IV at 1 dpi, nt = not collected, dpi = day post-infiltration.*

**FIGURE 5:**
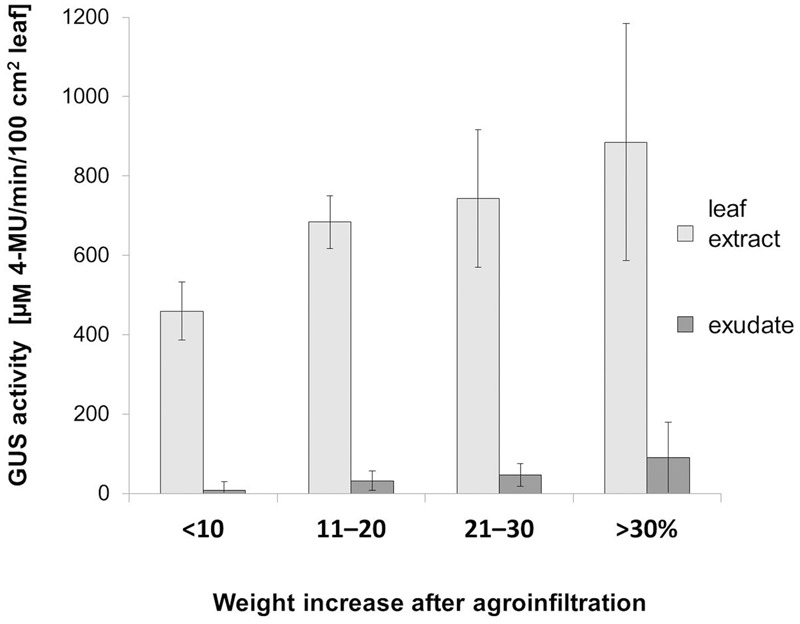
**Effect of agroinfiltration efficiency on the GUS activity in leaf blade and exudates.** To estimate the efficiency of bacteria introduction a leaf weight increase after infiltration was calculated as percentage values relative to the weight before the infiltration. We divided the infiltrated leaves (*n* = 14) into four classes (<10, 11–20, 21–30, and >30%) depending on the leaf weight (the X-axis). GUS activity (the Y-axis) in the whole leaf blades extracts as well as in the EDTA-exudate samples were measured at 1 dpi and then normalized relative to the 100 cm^2^ of leaf surface area [μM 4-MU/min in 100 cm^2^].

We then analyzed the effect of the bacteria introduction efficiency on the GUS activity in the transiently transformed leaves (**Table 2**, Experiment IV). The efficiency was calculated for each leaf as the percentage value indicating their weight increase after the infiltration (**Figure 5**). Depending on the increase of weight after the infiltration, the leaves were divided into 4 classes (<10, 11–20, 21–30, and >30%). The results showed that the GUS activity, both in the leaf extracts and in the EDTA-exudates, increased with the increase of the agroinfiltration efficiency.

## Discussion

The previous studies have established that only cucurbits in the early development stages can be successfully transformed using *Agrobacterium* while the mature plants are not susceptible to agrotransfection (Smarrelli et al., 1986; Nanasato et al., 2013; Ramírez-Ortega et al., 2014, 2015). However, our work has shown that *L. cylindrica* L. (*Cucurbitaceae*) can be successfully transformed by agroinfiltration.

One of the most important factors affecting agroinfiltration efficiency in several plant species is the susceptibility of their tissues to the introduction of bacterial suspension (Bhaskar et al., 2009; Leuzinger et al., 2013). *N. benthamiana*, whose leaves have excellent absorption properties enabling the optimization of the agroinfiltration procedure, has become the most popular transient expression system (Wydro et al., 2006; Goodin et al., 2008). Our analysis showed that among 11 species of cucurbits, only luffa leaves revealed an excellent absorption property. This absorption property allows the infiltration of the mesophyll tissue with relative large volumes of liquid containing high density suspensions of *Agrobacterium* cells.

The susceptibility of luffa leaves to infiltration can be explained by the loose structure of the mesophyll tissue with the large air spaces, especially revealed by the microscopic examination sub-stomatal air spaces. In contrast to luffa the mesophyll cells in other *Cucurbita* leaves are closely packed and the stomata air spaces are slight. High humidity promotes stomatal opening, which in consequence, increases access to the air spaces located in the deeper leaf tissues (Melotto et al., 2008; Kim et al., 2009). It was observed that high humidity conditions increase the agroinfiltration susceptibility of the luffa leaves. However, the compact structure of *Cucurbita* leaf tissues efficiently prevents the penetration of the externally applied bacteria into the deeper tissues despite the stomata opening. This could explain the low effectivity of transformation of *Cucurbita* plants.

The volume of intercellular spaces in leaves depends on plant species, leaf maturity as well as the growth conditions. Anatomical and physiological changes in leaves, in particular the reduction of intercellular space volume and thus the formation of more compact leaf tissue, were observed under abiotic and biotic stress in various plant species (Gausman et al., 1975; Beattie and Lindow, 1995). Luffa species are tropical and subtropical plants known as tolerant to various environmental stress conditions (Liao and Linn, 1996; Filipowicz et al., 2014; Li et al., 2014; Liu et al., 2016). The loose mesophyll structure in the leaves of luffa plants reported here indicates indirectly that the plants were grown under non-stress conditions.

Intercellular spaces and substomatal cavities in leaves provide also a habitat for endophytic phyllosphere microorganisms, especially for foliar pathogens (Beattie and Lindow, 1995). Foliar pathogenic bacteria can reach the leaf interior through natural surface openings (e.g., stomata, hydathodes) or through surface wounds (Wilson et al., 1999; Hirano and Upper, 2000). Numerous bacteria species use active mechanisms to colonize intercellular spaces, for example *Pseudomonas syringae* can use coronatine to actively open stomata (Melotto et al., 2008). Although, *A. tumefaciens* is a soil bacterium which infects underground plant organs it is commonly used as a tool for transformation of plant leaves (Cazzonelli and Velten, 2006; Goodin et al., 2008; Bhaskar et al., 2009; Leuzinger et al., 2013). Probably, it will be possible to increase the agroinfiltration efficiency using some of the colonization strategies described for foliar endophytes. One particularly interesting possibility will be to use different extracellular polysaccharides isolated from suitable *Pseudomonas* species as adjuvants to support the penetration of *Agrobacterium* into the interior of the leaves (El-Banoby and Rudolph, 1979).

Our results suggest the possibility to use luffa leaves as a tool for studies requiring the application of liquids into the leaf tissue. Such techniques are already used in plant physiology and biotechnology studies, especially for evaluating phloem functions in different plant species (Grignon et al., 1989; Pradel et al., 1999; Zhang et al., 2010; Knoblauch et al., 2015). In the case of cucurbits the problem with the application of liquid substances into mature leaves generally required some damage of leaf surface by using, e.g., fine sandpaper or carborundum as an abrasive (Grignon et al., 1989; Pradel et al., 1999; Shalitin and Wolf, 2000; Arazi et al., 2001; Zhang et al., 2010; Dunham et al., 2014; Kang et al., 2016). In contrast, the infiltration of luffa leaves is a more delicate method as it uses the natural ability of leaves to absorb water. The absorbance properties can be additionally elevated by pretreatment of luffa leaves in high humidity conditions.

The plant transformation techniques including transient transformation methods are used as a popular research tool for the elucidation of phloem functions and biochemistry in plant species known as apoplastic phloem loaders, such as *N. benthamiana, S. tuberosum*, or *A. thaliana* (Chincinska et al., 2008; Krügel et al., 2012). There are the distinct differences in the phloem structure between cucurbits and other plants (Turgeon and Medville, 2004). Although many species of *Cucurbitaceae* are widely used in research of phloem functions and biochemistry, only a few reports provide data obtained using transgenic cucurbits (Smarrelli et al., 1986; Nanasato et al., 2013; Ramírez-Ortega et al., 2014, 2015; Cheng et al., 2015). Many questions about cucurbit phloem functions (Slewinski et al., 2013), such as regulation of symplastic loading or long-distance movement of macromolecules, as well as phloem exudation and sealing mechanisms, have not been elucidated so far. The system for rapid and easy expression of foreign genes in mature luffa leaves provides an additional strategy for cucurbit phloem research.

It would be especially interesting to use the luffa leaves expression system as a tool for monitoring of macromolecules long distance trafficking in the phloem. Our observation indicates that GUS molecules can move from the agroinfiltrated leaf tissues, where they are produced, along the non-transformed petioles to the EDTA solution, in which the leaves were immersed after the infiltration. Phloem trafficking of recombinant proteins has already been described, but the mechanisms regulating these phenomenon are still not explained. For instance, Imlau et al. (1999) observed the long-distance trafficking of non-phloem 27 kDa GFP protein in transgenic *A. thaliana* and in tobacco plants expressing *GFP* driven by the AtSUC 2 phloem-specific promoter. The molecular weight of GUS protein in a monomeric form is 68 kDa and it is higher as GFP weight. Moreover, the GUS protein is active only in tetrameric form. Although, it was previously estimated that proteins greater than 200 kDa can move in the cucurbit phloem (Balachandran et al., 1997; Walz et al., 2004), it seems unlikely that the GUS protein is transported over long distances as an active tetrameric protein. This issue needs to be clarified, because the process of GUS tetramerization is reversible (Matsuura et al., 2011). We suppose that GUS can be transported as monomeric subunits, whose assembly into tetramers take place away from the protein production site.

The luffa leaves expression system can be used for a variety of cucurbit studies, including luffa itself. There are numerous reports on a wide range of luffa plants applications (Sujatha et al., 2013). It was shown that a number of substances identified in luffa seeds and leaf extracts has therapeutic potential, for example, a novel class of small molecule ribosome-inactivating peptides, which in addition to a strong inhibitory activity on cell-free protein synthesis show also antifungal, antimicrobial, and anti-tumorial properties (Ling et al., 1993; Parkash et al., 2002; Li et al., 2003). Presumably, transformed luffa leaves can become a more compatible expression system for the production of these luffa proteins (as well as other cucurbit-derived recombinant proteins) than expression systems based on bacteria or yeast (Liu et al., 2010).

The *L. cylindrica* leaves can be transiently transformed in a comparatively simple manner as in the case of *N. benthamiana* model plants. However, it can be assumed that the further optimization of the agroinfiltration procedure will lead to improvement of the expression system based on transiently transformed luffa leaves. It will be of particular interest to monitor the presence of recombinant proteins in exudate. Furthermore, the optimization of heterological expression in the luffa system enables the production and purification of recombinant proteins not only in transiently transformed leaves but also in exudates.

This study is the first case report about the application of cucurbits in the study of gene expression by agroinfiltration of mature leaves. The possibility to ease the genetic modification of mature luffa leaves will contribute to the development of our knowledge about *Cucurbitaceae* biology, especially in regards to the regulation of phloem functions. This will be the case not only in cucurbit research but also among scientists looking for a simple and reliable method for the heterologous expression in eukaryotic systems.

## Author Contributions

IC conceived the research plans. IC supervised the experiments. KB, MK, EZ, and IC performed the experiments and analyzed the data. IC conceived the project and wrote the article with contributions of all the authors. KB, MK, EZ, ZT, and IC supervised and complemented the writing.

## Conflict of Interest Statement

The authors declare that the research was conducted in the absence of any commercial or financial relationships that could be construed as a potential conflict of interest.
