# Supplementary material for: Mature Luffa Leaves (Luffa cylindrica L.) as a Tool for Gene Expression Analysis by Agroinfiltration
Source: Front Plant Sci. 2017 Feb 21;8:228. doi: 10.3389/fpls.2017.00228 (PMC5318407; doi:10.3389/fpls.2017.00228)
Supplement: Supplementary file 1 [file Table_1.DOCX]

Supplementary Material

Mature luffa leaves (Luffa cylindrica L) as a tool for gene expression analysis by agroinfiltration

Kamila Błażejewska1, Małgorzata Kapusta2, Elżbieta Zielińska1, Zbigniew Tukaj1, Izabela Chincinska1*

*** Correspondence:** Corresponding Author: i.chincinska@biol.ug.edu.pl

## Supplementary Table

## Supplementary Table 1. GUS activity in the agroinfiltrated luffa leaves and exudates.

| **GUS activity [µmol 4-MU/min/mg TSP]** | | | | | | | | | | | | |
| --- | --- | --- | --- | --- | --- | --- | --- | --- | --- | --- | --- | --- |
| **No. exp.** |  | | | **EDTA-exudates** | | | | | | | | |
|  | **Leaf extracts** | | | **1 dpi** | | | **2 dpi** | | | **3 dpi** | | |
|  | Mean | SD | N | Mean | SD | N | Mean | SD | N | Mean | SD | N |
| Exp. I | 41.7 | 30.6 | 6 | 105.0 | 63.5 | 6 | 105.1 | 63.6 | 6 | 16.6 | 5.0 | 6 |
| Exp. II | 127.6 | 76.5 | 10 | 151.7 | 107.6 | 4 | 32.1 | 18.2 | 4 | 55.3 | 78.3 | 4 |
| Exp. IV | 31.4 | 21.9 | 14 | 74.3 | 45.5 | 14 |  |  |  |  |  |  |

## GUS activity were measured fluorescently in leaves extracts and EDTA-exudates and then normalised to the total soluble protein content. In the Exps. I–II were the leaves extracts collected at 3 dpi, but in the Exp. IV at 1 dpi. In the Exps. I–III were the exudates collected at 1–3 dpi, but in the Exp. IV at 1 dpi only.
