# Supplementary figures and images for: Mature Luffa Leaves (Luffa cylindrica L.) as a Tool for Gene Expression Analysis by Agroinfiltration
Source: Front Plant Sci. 2017 Feb 21;8:228. doi: 10.3389/fpls.2017.00228 (PMC5318407; doi:10.3389/fpls.2017.00228)

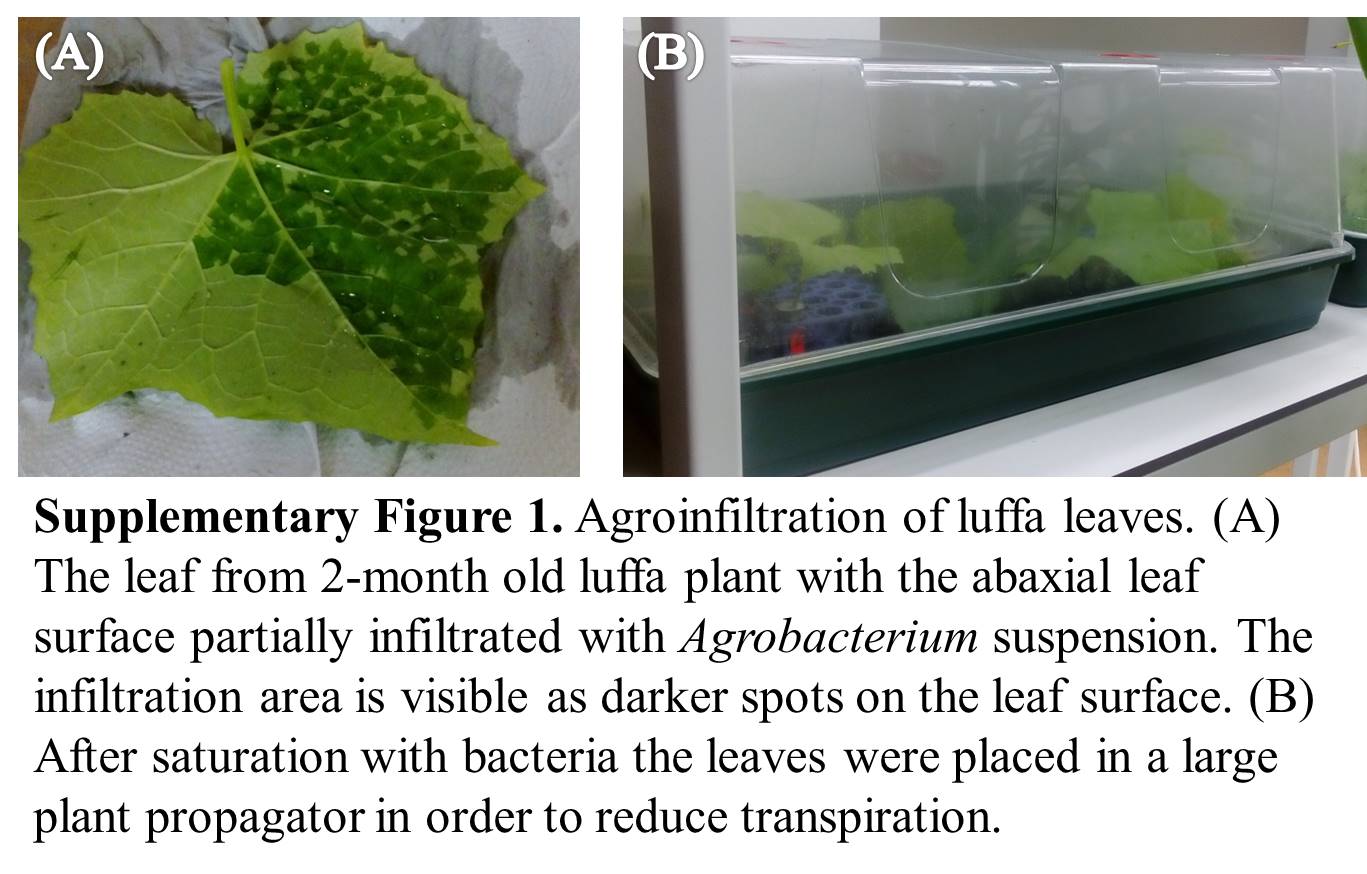

Supplement: Supplementary file 2 [file Image_1.JPEG]

S3. The histochemical X-gluc staining of GUS activity in luffa leaves at 1, 2 and 3 dpi

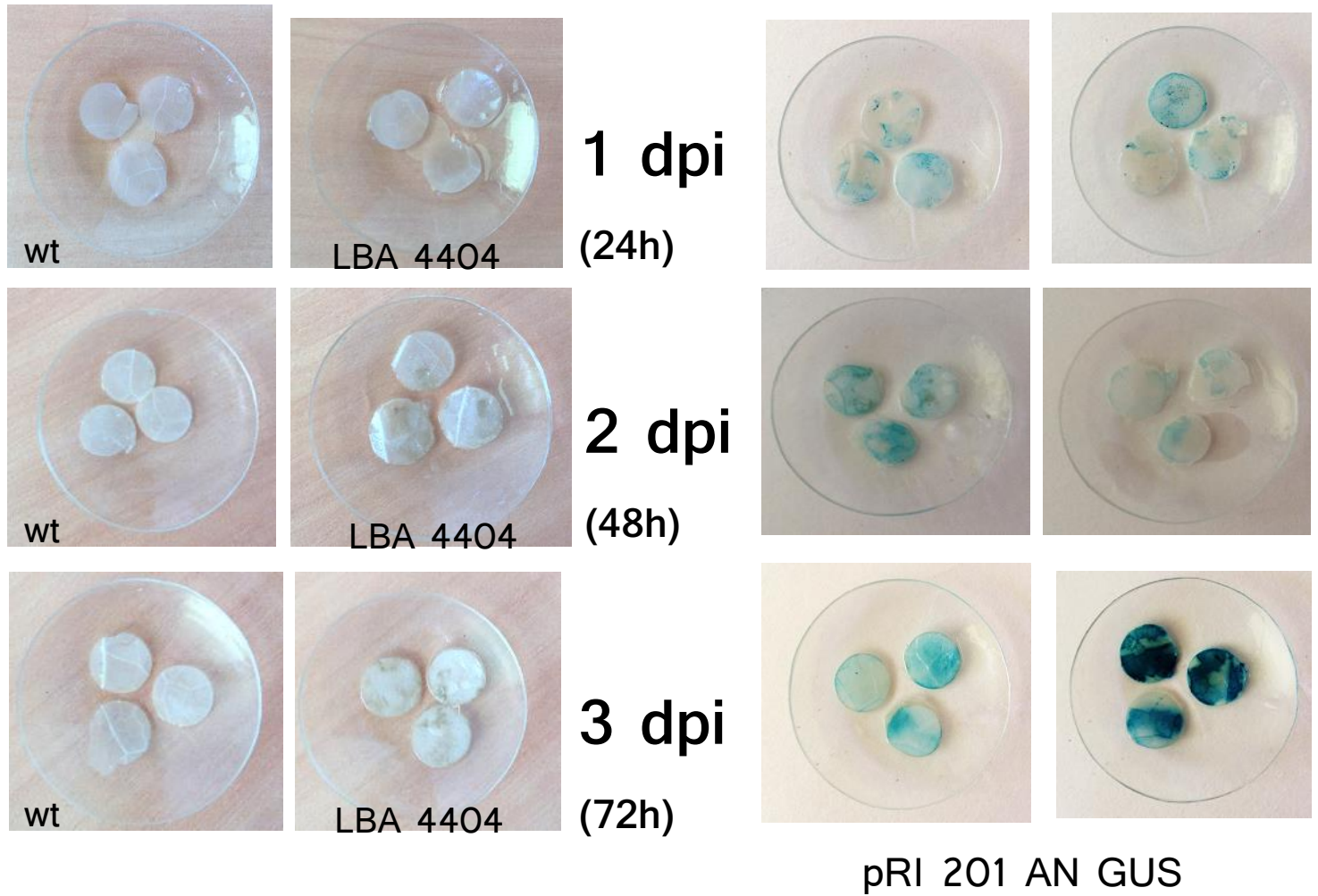

Supplement: Supplementary file 3 [file Image_2.PDF]
